# Material Selection Analysis of New Partial Discharge Sensor Electrode Plate Based on First-Principles Study

**DOI:** 10.3390/nano13030405

**Published:** 2023-01-19

**Authors:** Huiyuan Zhang, Zhensheng Wu, Fan Zou

**Affiliations:** 1 School of Electrical Engineering, Beijing Jiaotong University, Beijing 100044, China; 2 Department of Earth Science, Uppsala University, 62157 Visby, Sweden

**Keywords:** graphene, partial discharge sensor, electrode plate, first-principles, electrical transport

## Abstract

Graphene is well known for its electrical properties and can be used for sensor improvement. The first-principles study is one of the powerful tools to analyze and predict the performance of advanced materials. In this paper, microscopic material selection is performed for partial discharge sensor electrode plate materials based on first-principles study. By introducing graphene, six different microscopic electrode plate models are built based on the traditional metal electrode plates. Electrical properties including electronic structure, charge density and charge distribution of electrode plates are analyzed from the microscopic perspective when the actual partial discharge electric field is 1 V/m. Additionally, electrical transport properties of electrode plates are determined by electrical transport calculation. The results show that the double-layer graphene copper-clad electrode plate has better electrical transport capacity and higher gain characteristics when used in partial discharge sensors. This study fills the gap in the microscopic electric transport response mechanism of electrode plates, which can provide theoretical support for the improved design of partial discharge sensors.

## 1. Introduction

Transient earth voltage partial discharge sensors are widely used in the electrical field as non-invasive detection devices, which can be used to test the insulation status of electrical equipment [1]. With the development of power systems, digital grid transformation and miniaturization of electrical equipment, the insulation of electrical switchgear in the power systems may face many challenges [2]. In order to ensure the safe and stable operation of power systems, it is important to detect partial discharges [3]. It also puts forward higher requirements on the performance of partial discharge sensors [4]. Therefore, further improvement of partial discharge sensors is the key to improving the safe operation and reliability of power equipment [5]. Considering that the sensing principle is difficult to improve and the sensing structure is limited, the most practical strategy to improve the performance of partial discharge sensors is to improve sensing material. The electrode plate plays an important role in the transport of electrical signals as the core sensor piece. In order to improve the performance of the sensor, the material selection of the electrode plate must be studied. Therefore, the material selection of this paper is mainly oriented to electrode plate materials.

Graphene has carrier mobility up to 200,000 cm^2^/Vs. The resistivity of graphene is about 10^−6^ Ω/cm, which is smaller than that of silver, the lowest known resistivity [6,7]. It has become the first choice of sensing material in various sensors. In [8], a N/MEMS mechanical sensor with a graphene sensing element is proposed. In [9], a low-cost graphene-based interdigital capacitance pressure sensor is proposed. Ref. [10] proposed double-doped graphene as a gas sensing material for oxygen-containing gas molecules. However, in the field of transient earth voltage partial discharge sensors, no research based on graphene materials has ever been reported.

With the development of modern computing technology, the design of materials based on first principles has attracted the attention of researchers. First-principles study can not only help in understanding the electronically relevant mechanisms of materials, it can also reveal the intrinsic relationships of charge motion when computer simulations are more commonly used in the studies of material properties. In this way, it will obtain the microscopic mechanism of material properties, which can be used to guide experimental studies and even to predict novel materials. In [11], the mechanical and optical properties of hydrogenated RE_5_Si_4_ (RE = Sc and Y) were investigated, which provided theoretical support for the applications of rare-earth ultrahigh-temperature materials. In [12], the electronic structure and atomic-scale friction of graphene/ZrS_2_ heterostructures were also simulated, which played an important role in the design of new lubricant two-dimensional materials for reducing friction. In [13], the structural stability, electronic, optical, phononic, thermal properties and electronic transport coefficients of NaSrX materials were investigated; the study showed that NaSrX compounds are promising candidates for thermoelectric applications. In [14], the changes in total density of states (TDOS), partial density of states (PDOS), band gap (BG) and differential charge density (DCD) for NO_2_ adsorption by V-MoS_2_-WS_2_ were analyzed, which can be used to study the response mechanism of gas sensors prepared by two-dimensional heterojunction. The n*AlN/n*ScN superlattices were systematically studied in [15], which can be used as a paradigm in semiconductor superlattice electronic structure engineering for developing new dielectric film materials with high electrical energy storage. First-principles study allows the construction of materials at the atomic scale, which significantly reduces experimental costs and shortens the development cycles [16]. At the same time, combining microstructural parameters and macroscopic properties of materials from a microscopic perspective can reveal the mechanism of physicochemical properties of materials.

This paper studies microscopic material selection for transient earth voltage partial discharge sensor electrode plate materials based on first-principles study, considering that transient earth voltage partial discharge sensors are widely used in the electrical field as a non-invasive detection device. The electrode plate plays an important role in the transport of electrical signals as the core of the sensor. This study mainly focuses on the study of material selection for transient earth voltage partial discharge sensor electrode plates. The traditional partial discharge sensor electrode plate material is metal, which limits transport performance improvement. Considering the high conductivity and electron mobility of graphene materials, the transport performance of traditional partial discharge sensors can be effectively improved. Therefore, graphene is introduced on the basis of the metal electrode plate of traditional partial discharge sensors to carry out material selection research. Two kinds of metal electrode plates, copper and silver, which are common for traditional partial discharge sensors, are selected. Meanwhile, single-layer and double-layer graphene are introduced. By constructing microscopic models of six electrode plates and simulating the electric field of the partial discharge sensor, the electrical properties and electrical transport capacity of different sensing materials are compared. The calculation results of this work help to better understand the electrical properties and electrical transport properties of different electrode plates and even their similarities and differences. The transport gain characteristics of the macro partial discharge sensor can be further determined. It also provides some ideas for the design of corresponding advanced partial discharge sensing materials, which can be used to seek more valuable discoveries in electrical applications.

The rest of this paper is organized as follows. In Section 2, we introduce the principle of the transient earth voltage partial discharge sensor, density functional theory of the first-principles study, the non-equilibrium Green’s function and computational process, which are for our research; in Section 3, the microscopic model of six different electrode plates is built, and the details of the electrical properties and electrical transport calculations of the six electrode plates are given; in Section 4, the electrical properties of the six electrode plates are analyzed; in Section 5, the electrical transport of the six electrode plates is analyzed; finally, the main results and conclusions are presented in Section 6.

## 2. Computational Principle

### 2.1. Transient Earth Voltage Partial Discharge Sensor

Transient earth voltage partial discharge sensors are common sensors in the field of power system engineering. When partial discharge phenomenon occurs in the switchgear and other power equipment, electric charge will accumulate in the surrounding environment. Once the amount of charge reaches a certain level, it will cause a breakdown of electrical equipment, which seriously threatens the safety and stability of the power system.

The principle of transient earth voltage partial discharge sensors is electrical detection. The transient earth voltage partial discharge sensors electrode plate can couple partial discharge signals generated by electrical equipment to generate transient earth voltages. In this way, the partial discharge of electrical equipment can be judged.

The traditional transient earth voltage partial discharge sensor electrode plate material is ordinary metal. There is a problem of low sensitivity when used in the power system. For the partial discharge phenomenon in the actual environment, there are often errors such as untimely detection and misjudgment. Improvements to the transient earth voltage partial discharge sensor are needed. The traditional partial discharge sensor improvement mainly includes three aspects: principle improvement, structure improvement and material improvement. The principle is fixed as the transient earth voltage principle. In terms of structure improvement, the transient earth voltage partial discharge sensor has limited structural improvement due to the requirements of actual detection. Improving the material of the electrode plate for the transient earth voltage partial discharge sensor is a feasible approach. Therefore, it is necessary to consider the introduction of some practical materials, and the subsequent material selection based on the first-principles study was carried out to improve the transport performance of the partial discharge sensor.

### 2.2. First-Principles Study

The first-principles study is based on the basic principles of quantum mechanics. The electronic structure of materials is obtained by the method of quantum statistics through the mutual position and interaction of the nucleus and electrons of different substances. The physicochemical properties of materials are determined by their electronic structure, which in turn determines the various properties of the materials. In other words, the electronic properties of materials can be calculated from the first-principles study. The most important of these is the Schrödinger equation, and materials calculations are essentially solving the Schrödinger equation.

The first-principles study means that a semi-empirical treatment is used in the calculation of Schrödinger’s equation. It relies on the use of some analog quantities instead of empirical parameters. A multi-particle system consisting of atoms and nuclei is used to deal with polyatomic systems. Substances consist of atoms, in which the nuclei and electrons determine the properties of substance. Therefore, knowing the ground state of an atom, the wave function of the system can be obtained. In this way, its electronic properties are obtained. By solving the Schrödinger equation, all possible properties of materials can be obtained by applying it to first-principles study.

The Schrödinger equation is [17]:

(1) $$E\psi \left(\left\{{\vec{R}}_{I};{\vec{r}}_{i};t\right\}\right)=\hat{H}\psi \left(\left\{{\vec{R}}_{I};{\vec{r}}_{i};t\right\}\right)$$

where *E* is the energy eigenvalue, *ψ* and *Ĥ* are the wave function and Hamiltonian of the system, *R* represents the nucleus coordinates, *r* represents the electron coordinates and *t* represents time. The Hamiltonian of the multi-particle system includes the kinetic energy terms of all the particles in the solid and the interaction terms between the particles, without considering other external fields:

(2) $$\hat{H}=-\sum_{I}\frac{\hbar^{2}}{2M_{I}}\nabla_{{\vec{R}}_{I}}^{2}-\sum_{i}\frac{\hbar^{2}}{2m_{e}}\nabla_{{\vec{r}}_{i}}^{2}-\sum_{iI}\frac{Z_{I}e^{2}}{\left|{\vec{R}}_{I}-{\vec{r}}_{i}\right|}+\frac{1}{2}\sum_{ij(j\neq i)}\frac{e^{2}}{\left|{\vec{r}}_{i}-{\vec{r}}_{j}\right|}+\frac{1}{2}\sum_{IJ(J\neq I)}\frac{Z_{I}Z_{J}e^{2}}{\left|{\vec{R}}_{I}-{\vec{R}}_{J}\right|}$$

where *ℏ* represents the approximate Planck constant, *m_e_* represents the mass of the electron, *e* represents the elementary charge, *M_I_* represents the mass of the nucleus and *Z_I_* represents the number of charges carried by the nucleus. It can be abbreviated as:

(3) $$\hat{H}={\hat{T}}_{I}+{\hat{T}}_{i}+{\hat{V}}_{ion}+{\hat{V}}_{ii}+{\hat{V}}_{II}$$

where the first term is the kinetic energy of the nucleus, the second term is the kinetic energy of the electron, the third term is the interaction energy between the nucleus and the electron, the fourth term is the interaction energy between the electron and the nucleus, the fifth term is the interaction between the nuclei.

Since the Hamiltonian does not contain time, the variables can be separated to obtain the constant state Schrödinger equation [18]:

(4) $$\left[{\hat{T}}_{I}+{\hat{T}}_{i}+{\hat{V}}_{ion}+{\hat{V}}_{ii}+{\hat{V}}_{II}\right]\psi =E\psi$$

In general, all the properties of the system are contained in the solution-wave function of Equation (4). However, it is not very practical to solve the equation directly, especially for the multi-electron system. In practice, several approximations are generally used to solve it, such as the adiabatic approximation, Hartree–Fork method and density functional theory method (Hohenberg–Kohn theorem, Kohn–Sham equation). The density functional theory method is commonly used today.

### 2.3. Density Functional Theory

The concept of density functional theory (DFT) originated from the Tomas–Fermi model and it is developed on the basis of the Hohenberg–Kohn theorem. Calculations based on the Hartree–Fock principle allow predictions to be made for microscopic systems. It describes the system in terms of electron density distribution functions rather than wave functions. This is a huge simplification for multi-electron systems. Because of the time saving and efficiency, density functional theory is more widely used. After proposing the density functional theory in which electron density determines all the properties of molecules, it has led to increasingly deeper research in some fields, especially quantum chemistry. 

The formulation of the Hohenberg–Kohn first and second theorems laid a solid foundation for the density functional theory, whose main conclusions are as follows [19]:

(1) For a fermionic system without considering the spin, ground state energy E is the only generalized function of the particle density function *E*[*ρ*(***r***)].
(2) The ground state energy E of the system is equal to the minimum value taken at a constant number of particles n. According to these two theorems, the Hamiltonian quantity of the system can be defined as:

(5) $$\hat{H}=\hat{T}+\hat{V}+\hat{U}$$

where the first term is the kinetic energy of the electron, the second term is the external potential, which refers to the potential energy other than the electron interaction, such as the Coulomb potential of the nucleus, and the third term is the electron interaction potential.

The wave function of the system *ψ* can be uniquely determined from *Ĥψ* = *Eψ*, and the unique electron density is obtained. Kohn and Sham derived the Kohn–Sham equation by extracting the main parts of the kinetic energy of the electron and electron interaction potential and combining the rest to obtain the exchange–correlation term:

(6) $$\left(-\frac{1}{2}\nabla^{2}+v\left(r\right)+\int \frac{\rho \left(r^{\prime }\right)}{\left|r-r^{\prime }\right|}dr^{\prime }+v_{xc}\left[\rho \left(r\right)\right]\right)\varphi_{i}\left(r\right)=\varepsilon_{i}\varphi_{i}\left(r\right)$$

(7) $$\rho \left(r^{\prime }\right)={\sum_{i=1}^{N}\left|\varphi_{i}\left(r\right)\right|}^{2}$$

where ∇ is the kinetic energy operator, *v*(***r***) is the nuclear electron Coulomb potential, the third term is the electron Coulomb potential, *v_xc_*[*ρ*(***r***)] is the exchange–correlation potential, *ε_i_* is the Kohn–Sham energy eigenvalue. The Kohn–Sham equation can be solved by combining Equation (7) with the self-consistent field (SCF) method. It can be used to further solve the Schrödinger equation and thus, to predict the electrical properties of the material.

### 2.4. Nonequilibrium Green’s Function

For models whose dimensions are at the microscopic level, once the transport coefficients are found, then the transport characteristics of the system can be accurately obtained. The Green’s function approach relates the transport coefficients to the Green’s function of the system [20]. Therefore, for the microscopic system, Green’s function of the system can be defined on the basis of the exact definition of the transport channel mode of the system and the definition of the Hamiltonian quantity of the system. The electron transport coefficient is expressed as a function of Green’s function. Therefore, for the electron transport problem in the microscopic system, the Green’s function method can be used more conveniently.

Green’s function is a widely used method in physical research. Its core is to construct the equations of motion based on the Hamiltonian quantities of the system. The Green’s function represents the Hamiltonian of the system, and the basic information of the system, such as the density of states and the chance of leap, can be calculated by the Green’s function [21]. The problem of solving the wave function of the system is avoided and the electronic structure and transport properties of the system are obtained directly. Depending on the system to be treated, the Green’s function method is divided into two categories: equilibrium Green’s function (EGF) and non-equilibrium Green’s function (NEGF) [22]. When studying experiments, the structure of the material system under study is not in equilibrium due to the addition of electrodes. The traditional diagonalization method is no longer used, so the non-equilibrium Green’s function method is chosen to solve for the system [23].

The basic idea is to use DFT to calculate Hamiltonian quantities and electronic structures. The NEGF is used to calculate the electron density of the non-equilibrium system. Finally, the Landauer–Biittiker is used to calculate the bulk transport coefficient.

The Landauer–Biittiker formula is [24]:

(8) $$I(E)=\int_{\mu_{L}}^{\mu_{R}}T(E)\left(f_{L}\left(E-\mu_{L}\right)-f_{R}\left(E-\mu_{R}\right)\right)dE$$

where *μ_L_* and *μ_R_* are the chemical potentials of the left and right electrodes, respectively; *f_L_* and *f_R_* are the electron distribution functions of the left and right electrodes, respectively; *T*(*E*) is the transport coefficient of electrons with energy *E*, which can be calculated by the following equation [25]:

(9) $$T(E)=Tr\left[\Gamma_{L}(E)G^{R}(E)\Gamma_{R}(E)G^{A}(E)\right]$$

where *T_r_* represents the matrix tracing, *G^R^* and *G^A^* are the delayed and advanced Green’s functions of the intermediate scattering region, respectively; Γ*_L_* and Γ*_R_* are the coupling functions of the intermediate scattering region with the left and right electrodes, respectively. The transport coefficient *T*(*E*) can be considered as the sum of the transport coefficients of each of the intrinsic channels that do not intermingle with each other, as follows:

(10) $$T(E)=\sum_{n}T_{n}(E)$$

After calculating the transport coefficient of the model, its electrical transport capacity can be further determined.

### 2.5. Calculation Process

In this paper, Materials Studio 2019 computational simulation software is used for computational analysis, mainly using CASTEP and DMOL^3^ packages. First, a model of the atomic structure to be studied is built and the physical properties to be calculated are determined. The relevant calculation methods and parameters are set, such as the accuracy of the self-consistent field calculation, the size of the basic group and the k-point sampling density. In the calculation of the energy band structure of a crystal, the basis group represents the set of basis functions that are used to linearly combine into a single electron wave function. Before calculating the physical properties of the study object, the geometry of the constructed crystal structure model is first optimized to determine whether the crystal structure becomes stable (lowest energy) based on the convergence criteria of energy, displacement and stress. The physical properties of the geometrically optimized crystal are then calculated and the final results are output and analyzed. In this paper, we focus on the electrical properties and transport of the six electrode plates, which can be calculated from the optimized model. These results are related to the performance of the partial discharge sensor.

## 3. Computational Methodology

### 3.1. Computational Model

In the model building section, microscopic models of silver and copper are first created for metal electrode plates. Considering the subsequent introduction of graphene, a single-layer graphene model is created. Next, the AA stacked bilayer graphene model is formed by supercell with a layer spacing of 0.34 nm [26]. Regarding the way of introducing graphene for traditional metallic materials, considering that the composite of two substances is involved, heterojunction structure is used for construction in this paper [27]. Lattice mismatch degree is an important parameter for heterojunction structure. The single-layer graphene silver-clad electrode plate, single-layer graphene copper-clad electrode plate, double-layer graphene silver-clad electrode plate and double-layer graphene copper-clad electrode plate have lattice mismatch degrees of 4.1%, 15%, 4.1% and 15%, respectively. All of them satisfy the evaluation criterion of the lattice mismatch of maximum 25%, so lattice mismatch problems will not occur [28].

**Figure 1:**
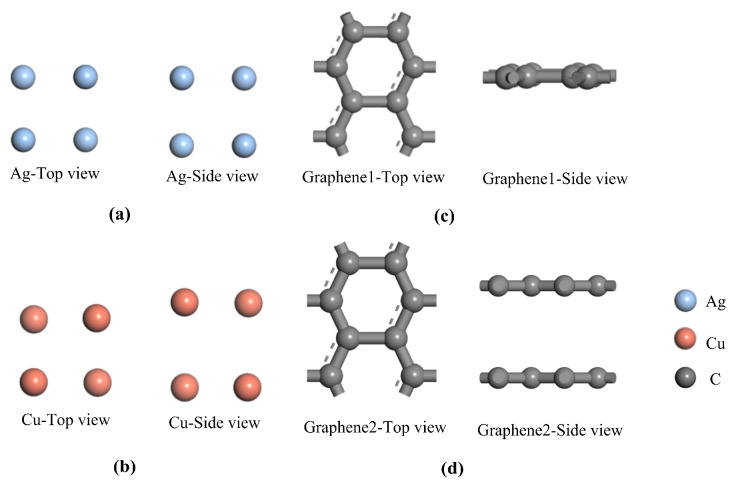
Optimized traditional metal electrode plate models and graphene models: (**a**) traditional silver electrode plate; (**b**) traditional copper electrode plate; (**c**) single-layer graphene; (**d**) double-layer graphene.

**Figure 2:**
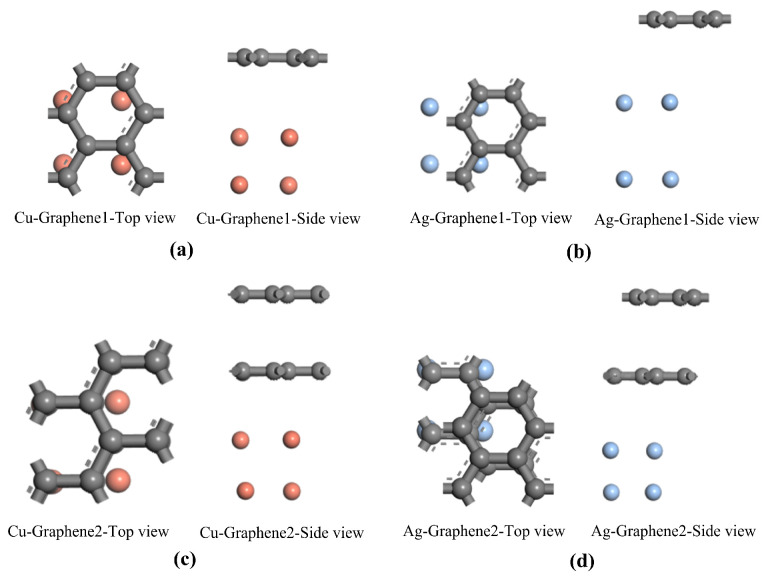
Optimized models of four composite electrode plates: (**a**) single-layer graphene copper-clad electrode plate; (**b**) single-layer graphene silver-clad electrode plate; (**c**) double-layer graphene copper-clad electrode plate; (**d**) double-layer graphene silver-clad electrode plate.

All electrode plate configurations are structurally optimized using the first-principles computational package CASTEP. The DFD-D scheme proposed by Grimme is used to correct for van der Waals forces [29]. As for the convergence criteria for the optimization, maximum atomic forces, maximum atomic forces and maximum atomic residual displacements are set as 0.002 eV/Å, 0.1 GPA and 0.002 Å, respectively. Self-consistent field (SCF) tolerance is set at 1 × 10^−4^ Ha. The parameters calculated for the studied microstructure are compared to determine whether the structure is stable or not and whether the convergence criteria are met. Finally, the six optimization models of this paper are finalized with the minimum energy as the benchmark shown in Figure 1 and Figure 2.

The two most stable microscopic structures of the optimized traditional metal electrode plates are shown in Figure 1a,b. The optimized single-layer and double-layer models of the introduced graphene are shown in Figure 1c,d. All of the optimized models above are consistent with the results of previous studies [30]. Using the optimized metal electrode plate models and graphene models in Figure 1, four composite models are further constructed including single-layer graphene silver-clad electrode plate, single-layer graphene copper-clad electrode plate, double-layer graphene silver-clad electrode plate and double-layer graphene copper-clad electrode plate. The four most stable optimized structures for the heterojunction formed by graphene and metal electrode plates are shown in Figure 2a–d. In general, in order to be able to study the physical properties of the subsequent system, the optimized model is used as a stable structure. The stability of the system can be ensured, which is an indispensable condition for the study of computational materials.

### 3.2. Calculation of Electrical Properties Based on DFT

The DFT-based electrical properties calculation can analyze and compare the charge motion and charge transfer capability of six different electrode plates in the partial discharge electric field. Additionally, the electrical properties calculation mainly revolves around the electron structure, charge density and charge distribution. In this way, the sequence of electron activities and electrical properties of different electrode plates can be further determined.

The electronic structure can be obtained by calculating the energy band structure and density of states. Among them, energy bands are used to qualitatively elucidate the general characteristics of electron motion in crystals. The size of the energy gap in the energy band diagram is determining whether the model belongs to a metal, a semiconductor or an insulator. This leads to further determining the electrical properties of the substance. The density of states (DOS) is essentially the number of different states that an electron is allowed to occupy at a given energy level. That is the number of electron states per unit volume of energy. In this way, the general distribution of states as a function of energy can be determined.

As for charge density, this paper focuses on differential charge density. It can be further used to analyze the charge transfer between atoms which helps to qualitatively analyze the difference in charge distribution. By calculating and analyzing the differential charge density, properties such as charge movement can be clearly obtained. However, charge transfer cannot be strictly quantified by the analysis of charge density.

The charge distribution is calculated using population analysis, which refers to the distribution of electrons in each atomic orbital. Mulliken charge population analysis can assign the electrons of the molecule to the basis functions in a certain way, and the charge distribution can be then analyzed quantitatively.

In this section of the electrical properties’ calculations, simulations are performed using the above six optimized electrode plates. Using the DMOL^3^ software package, the generalized gravity approximation (GGA) under the PBE generalized function is used to describe the exchange–correlation interactions [31,32]. The DFD-D scheme proposed by Grimme is used to correct for van der Waals forces [29]. The MIN basis is set in calculation. In addition, the self-consistent field (SCF) tolerance is set at 1 × 10^−4^ Ha. Brillouin zone is sampled by 1 × 1 × 1 k-points based on the Monkhorst–Pack method [33]. Meanwhile, in order to simulate the actual application environment of the partial discharge sensor, the electric field is set according to the actual partial discharge electric field of 1 V/m (2 × 10^−12^ a.u.; 1 a.u. = 5.142 × 10^11^ V/m).

### 3.3. Electric Transport Calculation Based on NEGF

**Figure 3:**
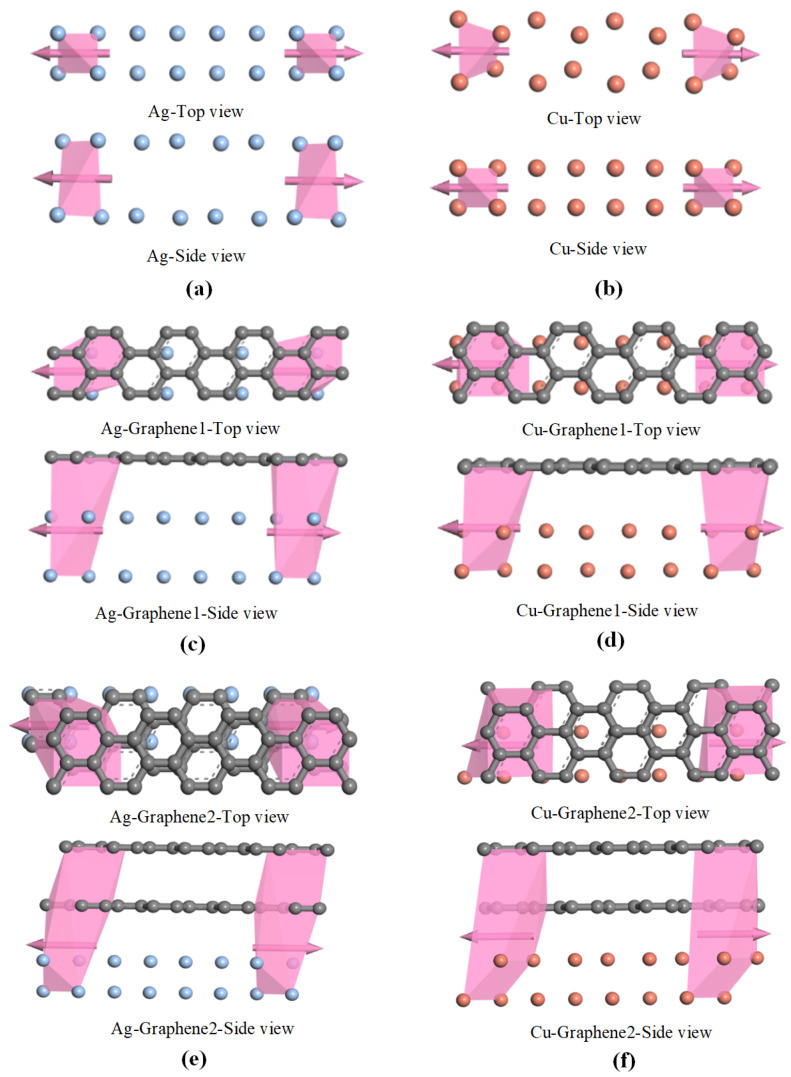
Transport models of six different electrode plates: (**a**) traditional silver electrode plate; (**b**) traditional copper electrode plate; (**c**) single-layer graphene silver-clad electrode plate; (**d**) single-layer graphene copper-clad electrode plate; (**e**) double-layer graphene silver-clad electrode plate; (**f**) double-layer graphene copper-clad electrode plate.

The calculation of electrical transport based on the non-equilibrium function is centered on the electrical transport capability of the material. The electrical transport capability will directly affect the transport gain of the partial discharge sensor, which is an important parameter to examine the detection capability of the partial discharge sensor. In this section, the transport curves of six different electrode plates are computed to determine the electric transport capacity. The electrical transport calculation process must begin with the construction of the transport model. The transport model is obtained by a 4 × 1 × 1 supercell on the basis of the optimized microscopic model in the previous section. The transport models of six different electrode plates are shown in Figure 3a–f.

The transport model contains three parts: the leftmost and rightmost parts are the two electrodes and the middle part is the intermediate scattering region. In order to control the variables in the simulation calculation and ensure that the model transport results are not disturbed by other factors during the calculation, the left and right electrodes of the electrode plates have the same structure as the middle scattering region. The left and right electrodes are connected to the intermediate scattering region as semi-infinite periodic materials, forming an open two-electrode system. The electron transport direction is the x-direction. Electrons enter from the left electrode to the intermediate scattering region area for scattering. One part of the electrons will be reflected back and the other part of them will be transported to the right electrode. Meanwhile, since the electrodes at both ends can be extended infinitely, the extension of the left and right electrodes can shield the influence of the intermediate scattering region on both electrodes.

Each extension has the same electronic structure as the connected electrodes and can be connected seamlessly without additional interface barriers. Then, some scattering energy points in the nonperiodic transport direction are selected and the wave functions of the left and right electrodes are solved using the Schrödinger equation, respectively. In this way, the scattering wave function and density matrix for the intermediate scattering region can be obtained. Therefore, the two electrodes only provide a reservoir of electrons for the intermediate scattering region of the system. The result is not affected by the changes in the intermediate scattering region and the boundaries.

The electric transport calculation consists of two steps: firstly, solving self-consistently for the electrodes at both ends, the effective potential energy can be obtained by superimposing all the occupied states according to the Fermi distribution function; secondly, solving self-consistently for the density matrix and scattering wave function in the intermediate scattering region using the effective potential energy at the boundary. The final transport coefficient curves for the six different electrode plates are derived before reaching the desired convergence accuracy.

In the calculation of electron transport, Dmol^3^ based on DFT and NEGF is used [31]. MIN basis set with DFT seminuclear pseudopotential (DSSP) is used [34]. The exchange correlation function is derived as generalized gradient approximation with Perdew–Burke–Ernzerh of (GGA-PBE). The Monkhorst–Pack grid k-point of 5 × 1 × 1 is applied in transport calculation [33]. In addition, the self-consistent field (SCF) tolerance is set at 1 × 10^−4^ Ha. In order to simulate the actual application environment of the partial discharge sensor, the electric field is set according to the actual partial discharge electric field of 1 V/m (2 × 10^−12^ a.u.).

## 4. Analysis of Electrical Properties

Considering partial discharge sensor electrode plates as an important component for coupling electrical signals, their electrical properties are very important. In order to compare and analyze the electrical properties of six different electrode plates, their electronic structures, charge densities and charge distributions are investigated from a microscopic perspective. The macroscopic electrical properties of six different electrode plate materials can be further understood by analyzing and comparing the microscopic electron activity sequences under actual discharge electric fields. The calculated electrical properties of the six electrode plate materials under a 1 V/m electric field are as follows:

### 4.1. Electronic Structure

**Figure 4:**
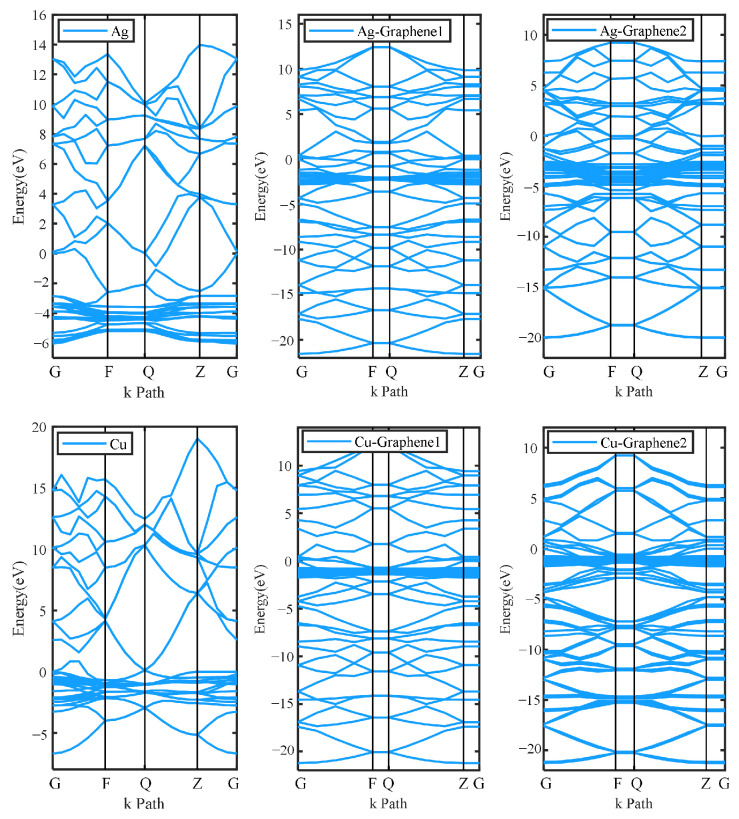
Energy band structure of different electrode plates.

The electronic structure can be determined by energy band and density of states analysis. It will further determine whether the six electrode plates are conductive or not. The energy band is shown in Figure 4.

Figure 4 shows that the energy band structure of the six electrode plates varies under the simulated partial discharge electric field. However, the band gaps are all zero and they all exhibit metallic properties, indicating that they all have the ability to conduct electricity. As partial discharge sensor electrode plates, all six electrode plates can couple the electrical signals generated during the actual discharge.

**Figure 5:**
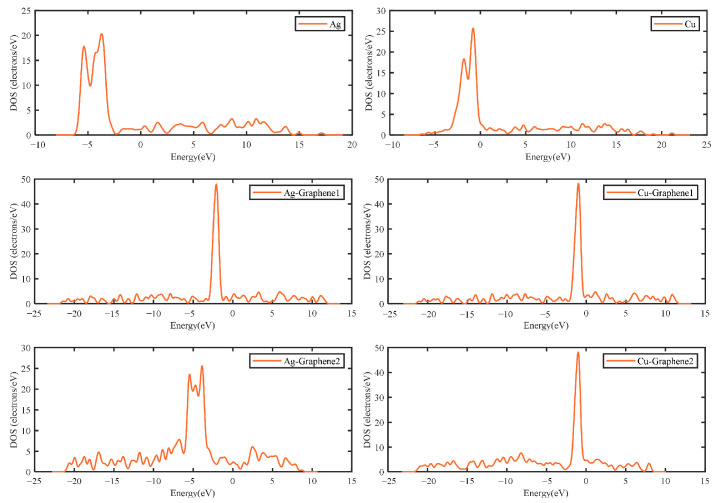
DOS of different electrode plates.

**Figure 6:**
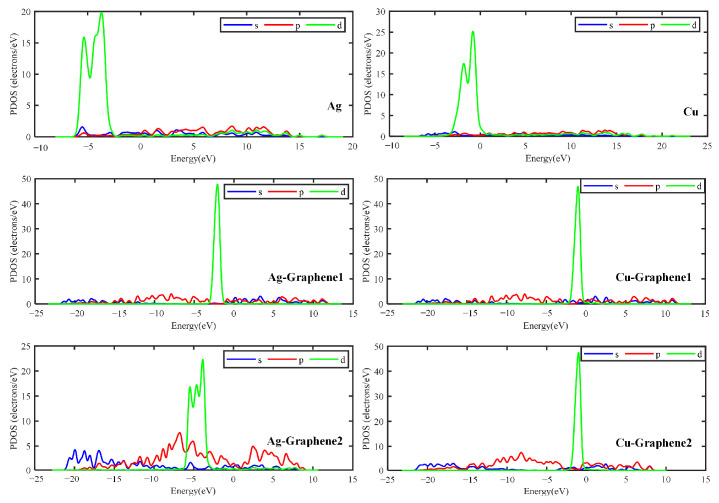
PDOS of different electrode plates.

As for the density of states, the total density of states and the partial density of states are listed separately for the six different electrode plate systems. The partial density of states reflects the potential distribution in different regions of the material. The total and partial density of states are shown in Figure 5 and Figure 6, respectively.

Figure 5 shows that the total DOS of the six electrode plates is not 0 near the Fermi energy level (energy = 0 eV) under the simulated partial discharge electric field. The non-0 DOS indicates that the electrode plates are metallic. This is consistent with the results from the energy band analysis. It is further verified that all six different electrode plates have the ability to conduct electricity and can be applied in partial discharge sensors. Figure 6 shows that the d orbitals of the six electrode plates contribute the most to the density of states. The contribution of s and p orbitals increases gradually with the increase of graphene layers. This is due to the fact that graphene is an sp^2^ hybrid structure and its introduction contributes to some extent to the DOS values of s and p orbitals. This is also consistent with the previous studies reported [30].

### 4.2. Charge Density

**Figure 7:**
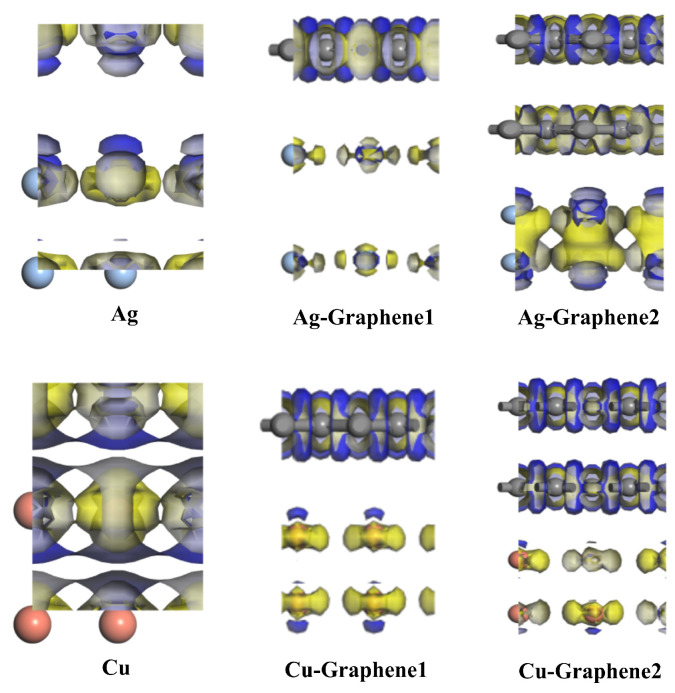
The three-dimensional differential charge density of different electrode plates.

In terms of charge density, the three-dimensions differential charge density of the six electrode plates and the two-dimensional differential charge density interface are shown in Figure 7.

Figure 7 shows that the yellow area represents electron deficiency and the blue area represents electron enrichment. According to the three-dimensional charge density distribution of the six different electrode plates, it can be seen that under the simulated partial discharge electric field, the charge density distribution of the two traditional metal electrode plates of silver and copper shows that both the electron gain and loss are in equilibrium. However, once graphene is introduced, the electrons of their metals in the constructed Ag-Graphene1, Cu-Graphene1, Ag-Graphene2, Cu-Graphene2 four composite electrode plates are in the electron-deficient state. Graphene shows electron enrichment. This indicates that the electron transfer from metal to graphene would have occurred after the introduction of graphene into the traditional metal electrode plate.

**Figure 8:**
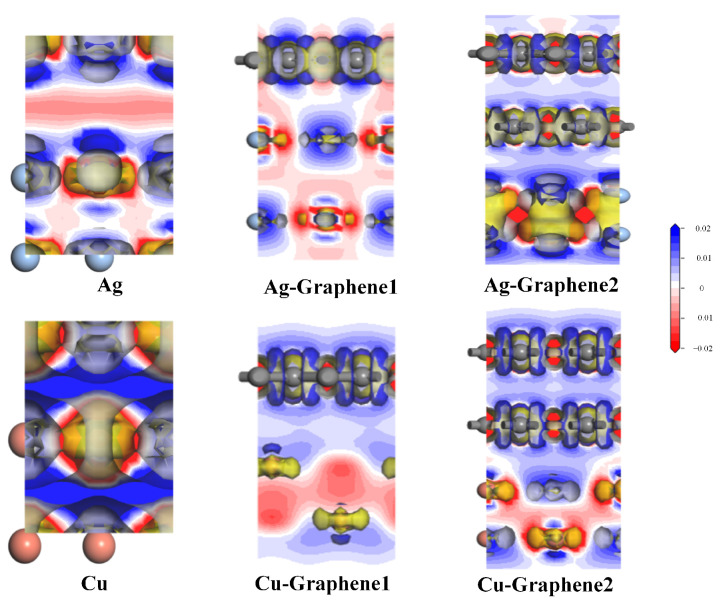
The two-dimensional differential charge density interface of different electrode plates.

In order to further analyze the charge transfer ability of different electrode plates and compare the transfer ability of six different electrode plates on the partial discharge signal after applying them to partial discharge sensor, the two-dimensional cross-sections are plotted. The differential charge density on the two-dimensional cross-sections are shown in Figure 8.

Figure 8 shows that the red area represents electron deficiency and the blue area represents electron enrichment. The lighter color means that charge transfer occurs in the electrode plates, showing greater interaction energy and charge transfer. It also shows that under the simulated partial discharge electric field, for both traditional metal electrode plates, the charge transfer capability of the silver electrode plate is higher than that of the copper electrode plate, i.e., Ag > Cu. This is consistent with the fact that the actual conductivity of silver is higher than that of copper. Once graphene is introduced, both graphene copper-clad electrode plates and graphene silver-clad electrode plates exhibit greater charge transfer. Meanwhile, the charge transfer ability of the copper electrode plate is significantly higher than that of the silver electrode plate after the introduction of graphene, i.e., Cu-Graphene1,2 > Ag-Graphene1,2. Moreover, increasing the number of layers will lead to a stronger charge transfer ability. In other words, for the transfer of partial discharge signals, the charge transfer ability of these four composite electrode plates are: Cu-Graphene2 > Cu-Graphene1, Ag-Graphene2 > Ag-Graphene1. The above analysis shows that Cu-Graphene2 has a stronger charge transfer ability and could be more suitable for practical partial discharge sensor electrode plate applications.

### 4.3. Charge Distribution

The charge density analysis is a qualitative analysis of charge transfer. To quantitatively analyze the charge distribution, the Mulliken charge population analysis calculations are performed. The charges obtained for each metal atom in the six different electrode plates are shown in Table 1 and Table 2.

Table 1 and Table 2 show that under the simulated partial discharge electric field, the traditional metal electrode plate is charge conserving with an external electron number of 0. Once graphene is introduced, charge transfer between graphene and metal will occur. The larger the charge value, the stronger the charge transfer capability will be. The charge relationship of the four composite electrode plates is Cu-Graphene2 > Ag-Graphene2 > Cu-Graphene1 > Ag-Graphene1. This result means that the more layers of graphene are introduced, the more electrons are on the metal atoms and the stronger the charge transfer ability. Meanwhile, for the same number of layers, it can be seen that the number of electrons on metal atoms in graphene copper-clad electrode plates is significantly higher than the number of electrons carried by metal atoms in graphene silver-clad electrode plates. In other words, graphene is more suitable for compounding with copper electrode plates to make composite materials, which is consistent with the results of the two-dimensional charge density analysis in Figure 8.

## 5. Electrical Transport Analysis

**Figure 9:**
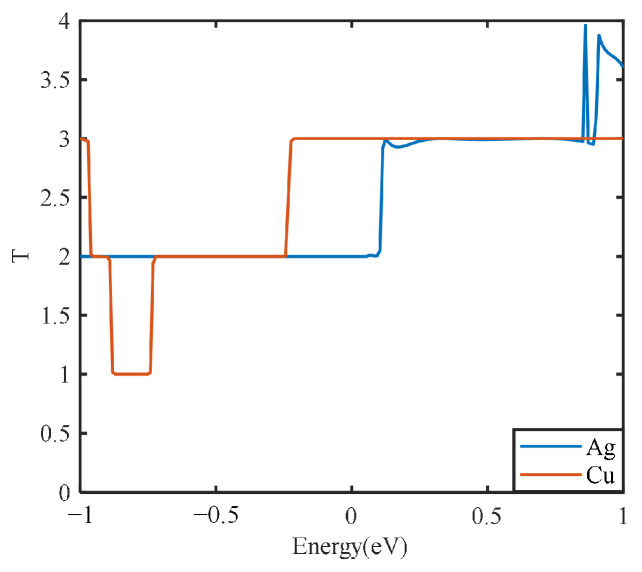
Transport curves of different metal electrode plates.

The electrical transport calculation can be used for further description of the electrical transport capacity of the electrode plate model in terms of the transport coefficient. The partial discharge transport capacity of different types of electrode plates under the actual discharge electric field can be characterized from a microscopic point of view. In this way, the effects of different metal types and different graphene layers on the electrical transport capacity of electrode plates can be further clarified. For both metal electrode plates, the transport coefficients are calculated from the microscopic perspective, and the results are shown in Figure 9.

Figure 9 shows that the integral areas of the transport curves of the silver and copper electrode plates are similar under the simulated partial discharge electric field. However, the peak value of 4 for the silver electrode plate is higher than that of the copper electrode plate. This is consistent with the actual conductivity of silver, which is consistent with the electrical property analysis described above. 

**Figure 10:**
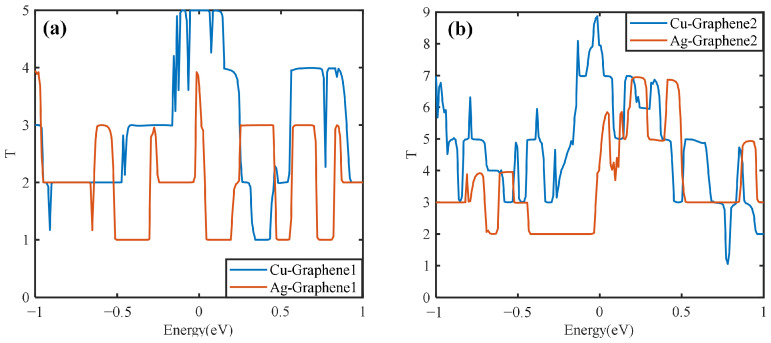
Transport curves of different metal and graphene composite electrode plates: (**a**) single-layer graphene copper-clad electrode plates and single-layer graphene silver-clad electrode plates; (**b**) double-layer graphene copper-clad electrode plates and double-layer graphene silver-clad electrode plates.

Graphene has excellent electrical conductivity, but when different metals are compounded with it, the transport properties will be different. Here, the electrical transport properties of single-layer graphene copper-clad electrode plates, single-layer graphene silver-clad electrode plates, double-layer graphene copper-clad electrode plates and double-layer graphene silver-clad electrode plates are calculated and compared. The effect of the metal type on the composite electrode plates has been explored and the results are shown in Figure 10.

Figure 10a shows that when a single layer of graphene is compounded with two different metals, the resulting graphene copper-clad electrode plate has a maximum transport coefficient of 5 under the simulated partial discharge electric field, which is higher than that of the graphene silver-clad electrode plate. It also has a much higher integral area than the graphene silver-clad electrode plate, i.e., Cu-Graphene1 > Ag-Graphene1. Figure 10b shows that the maximum value of the transport coefficient of the graphene copper-clad electrode plate is 9 when two layers of graphene are introduced on two different metals, which has a much higher integral area than the graphene silver-clad electrode plate i.e., Cu-Graphene2 > Ag-Graphene2. This indicates that when applied to the partial discharge sensor electrode plate, for the electrical signal in the electric field generated by partial discharge, the introduction of graphene on the copper electrode plate will have a stronger transport capacity than that on the silver electrode plate. It is consistent with the result from the electrical properties analyses.

**Figure 11:**
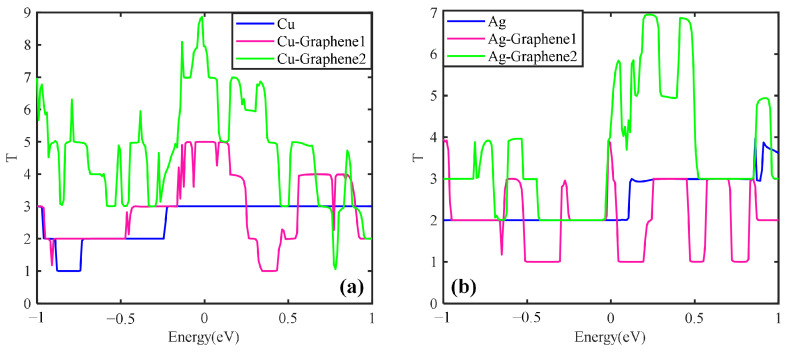
Transport curves of different metal and graphene composite electrode plates: (**a**) traditional copper electrode plate, single-layer graphene copper-clad electrode plate and double-layer graphene copper-clad electrode plate; (**b**) traditional silver electrode plate, single-layer graphene silver-clad electrode plate and double-layer graphene silver-clad electrode plate.

Meanwhile, the effect of graphene layers on the metal is analyzed and the transport characteristics of different graphene layers on the metal are studied. The results are shown in Figure 11.

Figure 11a shows that the transport coefficient increases with the number of graphene layers for the graphene copper-clad electrode plate under the simulated partial discharge electric field. When the number of graphene layers is 2, the peak value of the transport coefficient is 9 and the integral area is the largest, i.e., Cu-Graphene2 > Cu-Graphene1. Figure 11b shows that for the graphene silver-clad electrode plate, the transport coefficient increases with the increase of the number of graphene layers. When the number of graphene layers is 2, the transport coefficient peaks is 7 and the integral area is the largest, i.e., Ag-Graphene2 > Ag-Graphene1. It further shows that for the electrical signal in the partial discharge electric field, the introduction of double-layer graphene into the metal electrode plate will possess stronger electrical transport capability than the introduction of single-layer graphene, which is consistent with the conclusion of the electrical properties analyses.

## 6. Conclusions

For transient earth voltage partial discharge sensor electrode plates, this paper is based on first principles calculations of electrode plate materials for microscopic material selection. The electrical properties and electrical transport capacity of the new sensing materials under the actual electric field environment are studied. The method not only helps to understand the electron correlation mechanism of the materials, but also reveals the intrinsic relationship of the charge motion. It provides theoretical support for the development and application of electrode plate materials. It lays the foundation for the discovery and design of partial discharge sensing materials with excellent electrical transport capability.

(1) In terms of electronic structure, the six electrode plates have different energy bands, but the band gap is zero. They all exhibit metallic properties, indicating that all have the ability to conduct electricity.
(2) In terms of charge density, the charge transfer capability of the silver electrode plate is higher than that of the copper electrode plate. Once graphene is introduced, both graphene copper-clad electrode plates and graphene silver-clad electrode plates exhibit greater charge transfer. The charge transfer ability of the copper electrode plate is significantly higher than that of the silver electrode plate after the introduction of graphene.
(3) In terms of charge distribution, once graphene is introduced, the charge transfer between graphene and metal will take place. The number of electrons on metal atoms in graphene copper-clad electrode plates is significantly higher than the number of electrons carried by metal atoms in graphene silver-clad electrode plates.
(4) In terms of electrical transport, the transport coefficient of the silver electrode plate is higher than that of the copper electrode plate. When graphene is compounded with two different metals, the transport coefficient of the graphene copper-clad electrode plate is higher than that of the graphene silver-clad electrode plate.
(5) As for the number of layers, the higher the number of layers, the stronger the charge transfer ability and the higher the transport coefficient will be.

In summary, the first-principles study analysis for six different electrode plates shows that the double-layer graphene copper-clad electrode plate has better electrical transport capacity. That is, once the corresponding partial discharge sensor is prepared, it will have a higher gain characteristic. It is more suitable for the application of transient earth voltage partial discharge sensors. This theoretical calculation conclusion can be subsequently used to guide partial discharge sensor design and test. Moreover, this study only considers one layer and two layers graphene. In future work, we will further deepen the material design of the graphene partial discharge sensor from the micro aspects for the material selection using the first principle and experiments.

## Figures and Tables

**Table 1:** Charge distribution of three types of silver electrode plates (Unit: e).

Types	Ag1	Ag2	Ag3	Ag4	Total
Ag	0	0	0	0	0
Ag-Graphene1	0.005	0.005	−0.002	0.002	0.010
Ag-Graphene2	0.013	0.014	0.011	0.015	0.053

**Table 2:** Charge distribution of three types of copper electrode plates (Unit: e).

Types	Cu1	Cu2	Cu3	Cu4	Total
Cu	0	0	0	0	0
Cu-Graphene1	0.019	0.021	0.006	0.006	0.052
Cu-Graphene2	0.036	0.038	0.011	0.008	0.092

## Data Availability

Not applicable.
